# Sunitinib Treatment for Advanced Paraganglioma: Case Report of a Novel SDHD Gene Mutation Variant and Systematic Review of the Literature

**DOI:** 10.3389/fonc.2021.677983

**Published:** 2021-06-17

**Authors:** Franz Sesti, Tiziana Feola, Giulia Puliani, Roberta Centello, Valentina Di Vito, Oreste Bagni, Andrea Lenzi, Andrea M. Isidori, Vito Cantisani, Antongiulio Faggiano, Elisa Giannetta

**Affiliations:** ^1^ Department of Experimental Medicine, “Sapienza” University of Rome, Rome, Italy; ^2^ Neuroendocrinology, Neuromed Institute, IRCCS, Pozzilli, Italy; ^3^ Oncological Endocrinology Unit, Regina Elena National Cancer Institute, IRCCS, Rome, Italy; ^4^ Radiology Unit, “Santa Maria Goretti” Hospital, Latina, Italy; ^5^ Department of Radiological, Pathological and Oncological Sciences, “Sapienza” University of Rome, Rome, Italy; ^6^ Endocrinology Unit, Department of Clinical and Molecular Medicine, Sant’Andrea Hospital, “Sapienza” University of Rome, Rome, Italy

**Keywords:** sunitinib, paraganglioma, Familial Paraganglioma Syndrome, succinate dehydrogenase, SDHD

## Abstract

**Background:**

Paragangliomas (PGLs) are neuroendocrine neoplasms arising from chromaffin cells of sympathetic or parasympathetic paraganglia. Systemic therapies have been used only in metastatic PGLs. Antiangiogenic agents, such as sunitinib, could be a viable therapeutic choice in the subgroup of patients with *SDH*-positive PGLs. We describe the case of a man with Familial Paraganglioma Syndrome type 1 (FPGL) related to a novel mutation in *SDHD* gene treated with sunitinib. Furthermore, we performed a systematic review of the literature aimed to address the following question: is sunitinib treatment effective in patients with advanced/progressive/metastatic PGL?

**Methods:**

We performed a data search using MEDLINE, Cochrane Library, and Scopus between April 2019 and September 2020. We included studies reporting data on clinical or biological characteristics, or clinical outcomes of patients with PGLs treated with sunitinib.

**Results:**

The search leaded to the selection of 25 publications. Data from case reports and case series showed that disease control rate (DCR = stable disease + partial response + complete response) was achieved in 34.7% of cases under sunitinib treatment. In 39% of patients DCR was followed by progressive disease (PD) or tumor relapse, 26.1% patients showed PD. Data from clinical trials showed that DCR was 83%, and the median progression free survival was 13.4 months.

**Discussion:**

Data from the present literature review suggested that sunitinib could be a viable therapeutic option in advanced/progressive/metastatic inoperable PGLs. However, further trials on the efficacy of sunitinib in FPGL and sporadic PGL are needed.

## Introduction

Paragangliomas (PGLs) are neuroendocrine neoplasms (NENs) arising from chromaffin cells of sympathetic or parasympathetic paraganglia (1). A germline mutation is found in approximatively 40% of these tumors (2). Both in sporadic and inherited PGLs, it is possible to evidence two different pathogenetic pathways: alterations in proteins associated to Krebs cycle and hypoxia signaling (cluster I), and alteration in kinase signaling (cluster II) (3). Cluster I includes mutations in genes like von Hippel–Lindau (*VHL*), 2-oxoglutarate-dependent prolyl hydroxylase (*PHD2*), and succinate dehydrogenase (*SDH*), which is a component of the electron transport chain of the Krebs cycle and catalyzes the oxidation of succinate (4). Catalytic core of SDH is made of 2 subunits (SDHA, SDHB), anchored to the mitochondrial inner membrane through subunits SDHC and SDHD (5). Mutations in a subunit of SDH determine an accumulation of succinate, which causes an increase of hypoxia inducible factor (HIF) 1α for a reduction of its ubiquitination (6). The elevation in HIF1α leads to activation of the angiogenic pathway and alteration in cell metabolism (7).

*SDHD* mutations are responsible for Familial Paraganglioma Syndrome type 1 (FPGL1), inherited in autosomal dominant manner, characterized by head and neck parasympathetic PGLs (85% of cases), more rarely thoraco-abdominal sympathetic PGLs (20–25% of cases) and pheochromocytomas (10–25%) (8, 9). Some 157 mutations have been described in *SDHD* gene, including deletions and duplications (an updated list is available at https://databases.lovd.nl/shared/genes/SDHD) (10).

Malignant head and neck PGLs are extremely rare (8, 11). However, their local growth can determine compression and/or infiltration of the neighboring anatomical structures, causing dysphagia and cranial nerves palsy (12). Surgery represents the first-choice treatment for head and neck PGLs (13), but severe complications, including cranial nerve lesions and vascular damage can occur (14).

Systemic therapies have been used in metastatic PGLs only (15). Antiangiogenic agents, such as tyrosine kinase inhibitors (TKI), have demonstrated in a phase 2 trial to determine disease stability, as well as partial response in the subgroup of patients with SDH-positive PGLs (SDHA and SDHB) (16).

The effectiveness of sunitinib, a widely used TKI, in patients with advanced/progressive/metastatic PGL is still unclear. Starting from the observations in our clinical case, the present systematic review is aimed to address the following question: is sunitinib treatment effective in patients with advanced/progressive/metastatic PGL?

## Case Presentation

On September 2018, a 37-year-old man contacted the Neuroendocrine Tumor task force Unit (NETTARE) of the Policlinico Umberto I Hospital at “Sapienza” University for the appearance of bilateral later-cervical swellings three years before, diagnosed as PGLs. He smoked for 15 years. His personal history includes allergic asthma and coccygeal fistula surgically treated in 2003. The patient had a familial history for neoplasms on the father’s side: a grandmother with a non-better classified parathyroid neoplasm and a grandfather with a history of thyroid, bladder, and gastric cancer. On clinical examination left lateral cervical mass was fixed and firm, and no other relevant alterations were found. He reported dysphagia and dyspeptic symptoms without other disturbances; blood pressure and heart rate were normal.

The patient’s clinical history began in December 2015 when, for the appearance of bilateral laterocervical masses, he performed a magnetic resonance (MR) imaging of the neck that highlighted “the presence of two expansive lesions localized bilaterally in the vascular spaces of the neck, on the right of 45 × 25 × 70 mm and on the left of 48 × 44 × 53 mm, with an inhomogeneous architecture, and a marked and inhomogeneous contrast enhancement”. The MR angiography confirmed the vascular nature of the masses. The right lesion extended from the carotid bifurcation growing cranially up to the lacerated foramen, determining encasement of the internal and external carotid arteries, located both anteriorly. This formation exerted an evident mass effect in the retro-stylous vascular space, causing the lifting of the muscular tent of the upper constrictor and the partial distortion of the oropharyngeal air column. The left lesion surrounded the middle distal tract of the common carotid artery, determined encasement of two branches at the carotid bifurcation, but involved only the cervical tract of the internal carotid artery. Biochemical assessment showed negative urinary metanephrines (44 µg/24 h, range 20–345).

After two different multidisciplinary consultations, on September 2016 the patient underwent the removal of the right lesion after embolization, with histological examination compatible with PGL: “neoformation of 44 mm, consisting of solid round nests of monomorphic cells of medium-sized, immunoreactive for chromogranin A, synaptophysin, vimentin, negative for EMA, CAM5.2, AE1/AE3. At the periphery, cellular component of dendritic aspect, S100 positive. Ki67 index <2%”.

After surgery, the patient reported a lesion of the right hypoglossal nerve, developing right vocal cord hypo-mobility, paresis of the palatine veil on the right side, right hemilingual hypotrophy, and a complete deficit of tongue mobility. In March 2017, the left lesion was embolized.

**Figure 1:**
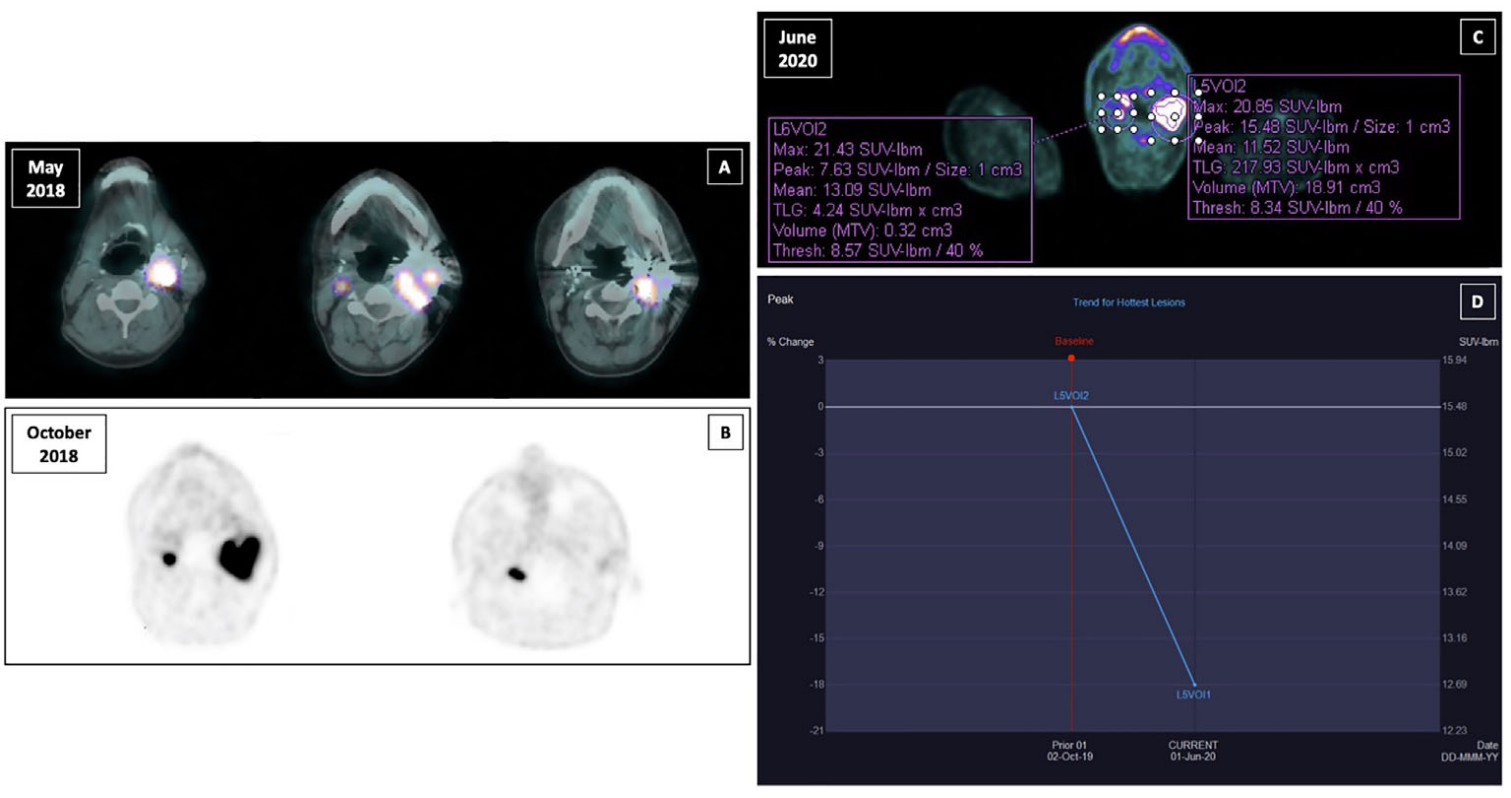
Functional imaging of right and left paraganglioma and response to sunitinib treatment assesed by PERCIST criteria. **(A)** 18F-fluoro-dihydroxyphenylalanine (18F-DOPA) positron emission tomography (PET)-computed tomography (CT) in May 2018; **(B)** 68Ga-DOTA-D-Phe1-Tyr3-Octreotate (68Ga-DOTATOC) PET-CT in October 2018; **(C)** 18F-fluorodeoxyglucose (18F-FDG) positron emission tomography (PET)-computed tomography (CT) with contrast medium performed after the third cycle of therapy in June 2020; **(D)** peak standardized uptake value corrected for lean body mass (SULpeak) trend for the hottest lesion between the two susequent 18F-FDG PET-CT (October 2020 and June 2020).

During the follow-up the patient performed several morphological and functional imaging exams. The MR and computed tomography (CT) angiography confirmed the presence of residual tissue on the right side of 15 mm, whereas the embolized lesion on the left appeared slightly reduced in size compared to the pre-operative examination (40 × 43 × 53 mm), with presence of vital tissue. Both lesions showed significant uptake of radiotracer at ^18^F-fluoro-dihydroxyphenylalanine (^18^F-DOPA) positron emission tomography (PET)-CT with a SUVmax of 10.5 on the left and 5.66 on the right, and at ^68^Ga-DOTA-D-Phe1-Tyr3-Octreotate (^68^Ga-DOTATOC) PET-CT (**Figures 1A, B**). Moreover, the latter showed a further area of focal uptake in correspondence of the skull base on the right, adjacent to surgical clips.

Considering the expression of somatostatin receptors, in December 2018 a therapy with somatostatin analogs (SSAs) was started (lanreotide 120 mg every 28 days subcutaneously). The patient experienced poor tolerance because of abdominal pain and acholic feces, so the dose was reduced to 90 mg every 28 days until the disappearance of the symptomatology. At 4-month MR angiography, tumor growth was observed (the right measured 22 *vs*. 15 mm; the left 69 × 46 × 50 *vs*. 40 × 43 × 53 mm). A ^18^F-fluorodeoxyglucose (FDG) PET-CT was performed according to our proposed follow-up algorithm (17), with the evidence of an intense metabolic activity in correspondence of the left voluminous expansive lesion (SUVmax 26.6) and the known right nodular lesion (SUVmax 27.4).

Given the morpho-functional features of the lesion (local aggressiveness, progression, high ^18^F-FDG uptake), the risk-effectiveness of the treatments’ strategies (high surgical risk, potential ineffectiveness of radiotherapy due to high tumor volume, poor efficacy and tolerance of SSA administration), and patient preferences, the NETTARE multidisciplinary board proposed a targeted therapy with a TKI: sunitinib.

**Figure 2:**
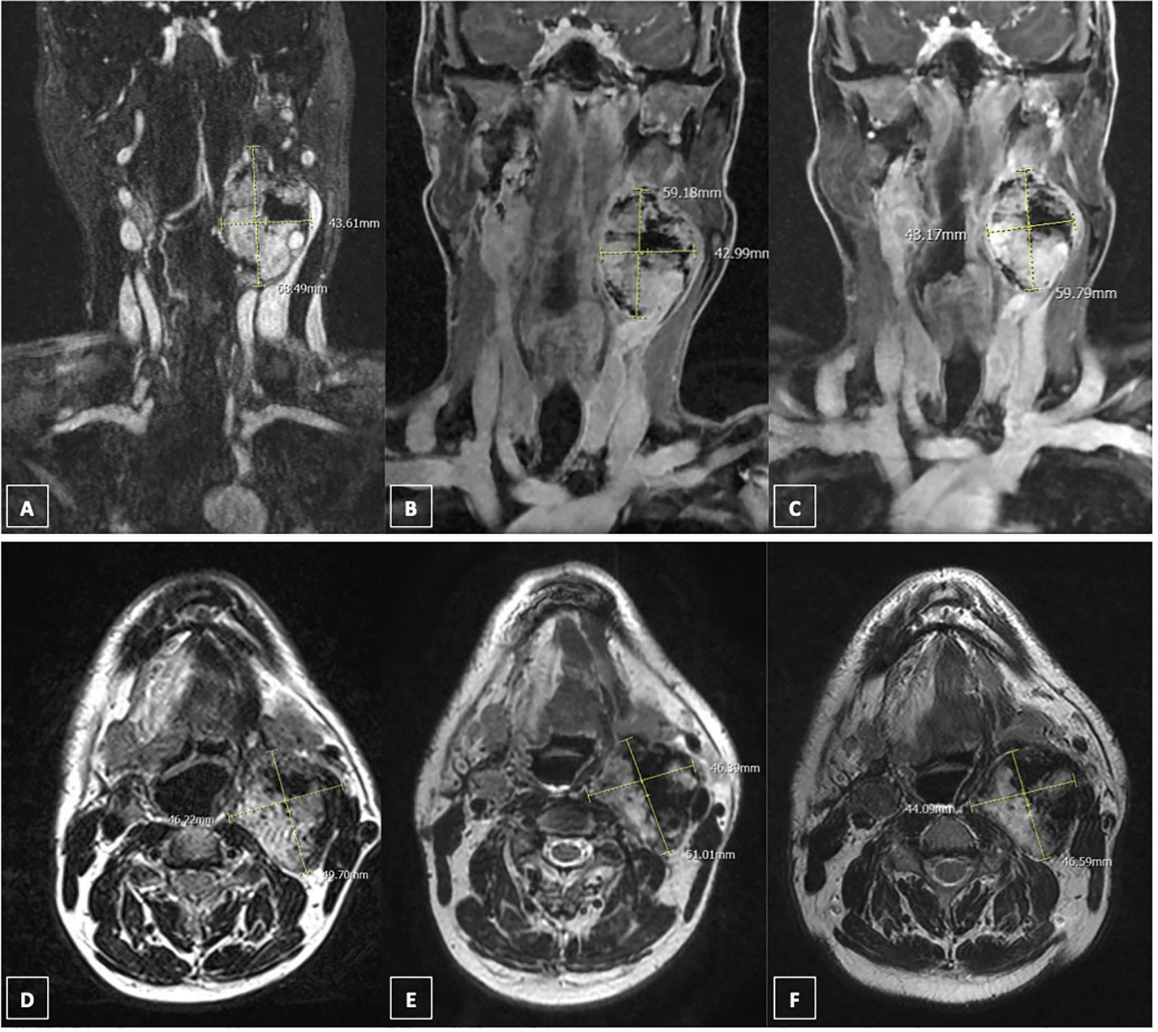
Response to sunitinib treatment assesed by magnetic resonance (MR). **(A)** coronal contrast-enhanced MR scan (fat-sat T1 sequence) of left paraganglioma in April 2019; **(B)** coronal contrast-enhanced MR scan (fat-sat T1 sequence) of left paraganglioma in July 2020; **(C)** coronal contrast-enhanced MR scan (fat-sat T1 sequence) of left paraganglioma in March 2021; **(D)** axial contrast-enhanced MR scan (T2 sequence) of left paraganglioma in April 2019; **(E)** axial contrast-enhanced MR scan (T2 sequence) of left paraganglioma in July 2020; **(F)** axial contrast-enhanced MR scan (T2 sequence) of left paraganglioma in March 2021.

In October 2019, sunitinib was started at a low dosage (25 mg daily orally), in order to minimize the potential toxicity, obtaining a relevant clinical response, with improvement of dysphagia and pain, and consequent improvement of quality of life. A good safety profile was reported. The follow-up with contrast-enhanced ultrasound (CEUS) of the neck showed a precocious size reduction of the lesions with onset of necrosis signals. The left lesion was decreased from 57 × 48 mm at baseline to 47 × 31 mm at 20 days and then was stable at 50 days, the right lesion was decreased from 13 mm at baseline to 9 mm at 20-day follow-up and then stable at 50-day follow-up. Near the end of the third cycle of sunitinib a ^18^F-FDG PET-CT with contrast medium was performed. The peak standardized uptake value corrected for lean body mass (SULpeak) of the left lesion was reduced by 18% (**Figures 1C, D**). Subsequently, a MR angiography performed at one-year follow-up showed the right lesion decreased to from 16 × 22 × 11 mm to 19 × 13 × 20 mm and left lesion decreased from 69 × 46 × 50 mm to 59 × 46 × 51 mm. At last MR follow-up in March 2021, right and left lesions were stable measuring respectively 18 × 16 × 13 mm and 60 × 44 × 47 mm. Thus, under sunitinib treatment the patient has been stable for 17 months (**Figure 2**).

Previously, the patient underwent a genetic consultation with the detection of a new mutation of *SDHD* gene c.16_28del p. Arg6PHEFS*5, that has never been described in literature, consistent with the diagnosis of FPGL1. A complete family history was collected in spite of the absence of other familial cases of PGL. At the genetic test among the first-degree relatives, the brother was negative, while the father was *SDHD* mutated. He underwent a neck and chest CT, that showed a lesion on the left carotid (max diameter 24 mm) as well as an enormous lesion in the mediastinum (>100 mm of maximal diameter), both with marked contrast enhancement. The ^68^Ga-DOTATOC PET-CT and ^18^F-FDG PET-CT were performed, highlighting uptake of both the radiotracers. The mediastinum lesion was removed after embolization by an expert team of thoracic surgeons of our Unit. The histological diagnosis was consistent with PGL: neoformation of 100 × 90 × 40 mm, associated to another neoformation of 15 mm with the same characteristics in proximity of the mail lesion. Both neoformations consisted of organoid nests of medium-sized cells with a large clear cytoplasm and a dispersed chromatin oval nucleus, which in the smaller lesion is hyperchromatic and irregular, surrounded by a thin continuous line of spindle cells with an elongated nucleus, and separated by a dense capillary vascular network. Immunochemistry was positive for synaptophysin, CD56, S100, GFAP, and weakly positive for chromogranin A. Ki67 index was 4%.

## Methods

A systematic review was performed following a rigorous protocol based on the Cochrane Collaboration and PRISMA statements (18, 19), in order to address the following question: is sunitinib treatment effective in patients with advanced/progressive/metastatic PGL?

English-language original articles were independently searched by one author (FS) in several databases (MEDLINE, Cochrane Library, and Scopus) between April 2019 and September 2020. The following key words were used for study search: (sunitinib AND paraganglioma) OR (sunitinib AND paraganglion tumor) OR (sunitinib AND paraganglionic tumor) OR (sunitinib AND paraganglion neoplasm) OR (sunitinib AND paraganglionic neoplasm). Additional articles were identified by hand-searching reference lists of all the eligible articles retrieved.

The titles and the abstracts of all identified articles were independently screened by one reviewer (FS) to assess their relevance. Reviews, animal studies and non-original articles were excluded. Full texts of selected, potentially relevant, papers were further evaluated. For the purpose of this review suitability of studies was defined as eligible if reporting data on the clinical or biological characteristics, or clinical outcomes of patients with PGL treated with sunitinib. We therefore selected studies that met all the following eligibility criteria: (i) randomized-controlled trial, prospective or retrospective studies, case series or case reports; (ii) PGL diagnosis; and (iii) treatment with sunitinib.

One author (FS) independently extracted the following data from included publications: first author, year of publication, study design, age, sex, primary tumor site, mutation status, sunitinib schedule, radiographic response criteria, radiographic response, response duration, and toxicity. **Table 1** summarizes these data.

**Table 1:** Response to sunitinib in patients from 25 selected case reports, case series, and clinical trial.

Author and year	Sex	Age at diagnosis	Primary tumor site	Primary tumor dimension	Catecholamine excess	Mutation status	Previous therapy	Sunitinib schedule	Radiographic response criteria	Radiographic response	Response duration	Toxicity
Joshua, 2009 (20)	M	55	Abdominal	14.3 cm	No	*SDHB*	None	50 mg daily for 4 weeks on, 2 weeks off (before surgery); 37.5 mg daily for 4 weeks on, 2 weeks off (after surgery)	RECIST	PR followed by PD	24 weeks	Neutropenia, plantar-palmar erythema, fatigue, hypertension
	M	28	Pelvic	7 cm	Yes	*SDHB*	Surgery, radiotherapy, cisplatin, etoposide	50 mg daily for 4 weeks on, 2 weeks off	RECIST	PR	40 weeks	Mild anorexia, diarrhea, hypothyroidism
Hahn, 2009 (21)	F	33	Abdominal	17 cm	NA	*SDHB*	Surgery, radiotherapy, E7974 (microtubule inhibitor), and paclitaxel	50 mg daily for 4 weeks on, 2 weeks off; 50 mg daily for 2 weeks on, 1 week off	NA	PR followed by PD	16 weeks	NA
Cirillo, 2010 (22)	M	37	Abdominal	17 × 14 × 9 cm	NA	NA	Surgery, doxorubicin, cyclophosphamide, vincristine and dacarbazine (modified CYVADIC), radiotherapy, ^131^I-MIBG, vinorelbine, octreotide, thalidomide	50 mg daily for 4 weeks on, 2 weeks off; 25 mg daily for 4 weeks on, 2 weeks off; 25 mg daily for 2 weeks on, 1 week off	NA	SD followed by PD	10 months	Hematuria, fever, abdominal cutaneous herpes, oral candidiasis, depressive syndrome, hypothyroidism
Zukauskaite, 2011 (23)	M	31	Head & Neck	10 × 15 cm	No	Sporadic	Cyclophosphamide, doxorubicin and vincristine, surgery, PRRT	50 mg daily for 4 weeks on, 2 weeks off	NA	SD followed by PD	24 weeks	Fatigue, rash, neutropenia
Ayala-Ramirez, 2012 (24)	NA	55	NA	NA	Yes	*SDHB*	Chemotherapy	50 mg daily for 4 weeks on, 2 weeks off or 37.5 mg daily continuously or 37.5 mg daily for 3 weeks on, 1 week off	RECIST	SD	27 months	NS (Hypertension, diarrhea, hand-foot syndrome, sore mouth, fatigue, elevations of serum creatinine)
	NA	20	NA	NA	Yes	*SDHB*	Chemotherapy	50 mg daily for 4 weeks on, 2 weeks off or 37.5 mg daily continuously or 37.5 mg daily for 3 weeks on, 1 week off	RECIST	SD	36 months	NS (Hypertension, diarrhea, handfoot syndrome, sore mouth, fatigue, elevations of serum creatinine)
	NA	45	NA	NA	No	*SDHB*	Chemotherapy, ^131^I-MIBG	50 mg daily for 4 weeks on, 2 weeks off or 37.5 mg daily continuously or 37.5 mg daily for 3 weeks on, 1 week off	RECIST	PR	4.5 months	NS (Hypertension, diarrhea, handfoot syndrome, sore mouth, fatigue, elevations of serum creatinine)
	NA	40	NA	NA	Yes	*SDHB*	Chemotherapy, ^131^I-MIBG	50 mg daily for 4 weeks on, 2 weeks off or 37.5 mg daily continuously or 37.5 mg daily for 3 weeks on, 1 week off	RECIST	SD	8 months	NS (Hypertension, diarrhea, handfoot syndrome, sore mouth, fatigue, elevations of serum creatinine)
Bourcier, 2013 (25)	F	70	Abdominal	NA	No	NA	Octreotide, surgery	50 mg daily for 4 weeks on, 2 weeks off	RECIST	CR	7 months	Hypotension
Prochilo, 2013 (26)	F	35	Abdominal	NA	No	*SDHB*	Surgery	50 mg daily for 4 weeks on, 2 weeks off; 37.5 mg daily continuously; 25 mg daily for 2 weeks on, 1 week off	NA	PR followed by SD followed by PD	9 months	Hypertension
Gillon, 2014 (27)	M	49	Abdominal	NA	Yes	NA	Surgery	50 mg daily, 4 weeks on, 2 weeks off; 37.5 mg daily continuously	RECIST	PR followed by PD	16 months	NA
Makis, 2016 (28)	F	22	Abdominal	14 cm	NA	*SDHB*	Chemotherapy, ^131^I-MIBG	50 mg daily for 4 weeks on, 2 weeks off	NA	CR followed by relapse	9 months	Gastrointestinal bleeding
Jeevan, 2016 (29)	F	77	Head & Neck	2 × 3 × 3 cm	No	NA	Surgery, radiotherapy	NA	NA	PR	24 months	NA
Belgioia, 2016 (30)	F	53	Head & Neck	NA	NA	NA	Surgery, radiotherapy, PRRT	50 mg daily continuously; 25 mg daily continuously	NA	PD	10 months	Mucositis and fatigue
Canu, 2017 (31)	M	35	Abdominal	4.6 × 4.9 × 5.9 cm	Yes	*SDHB*	Surgery	25 mg daily for 2 weeks on, 1 week off	RECIST	PR followed by SD followed by PD	77 weeks	None
Patel, 2017 (32)	M	47	Abdominal	10.8 cm	Yes	*SDHB*	Surgery	37.5 mg daily continuously	NA	PR followed by PD	12 months	NA
Ferrara, 2017 (33)	F	54	Abdominal	45 cm	Yes	*MAX*	None	37.5 mg daily continuously	RECIST	SD	4 months	Palmar-plantar erythrodysesthesia syndrome
Ong, 2018 (34)	F	51	Abdominal	NA	Yes	*SDHC*	Surgery, radiotherapy, octreotide, everolimus	NA	NA	PD	NA	NA
Stigliano, 2018 (35)	M	55	Pelvic	7 cm	No	*SDHB*	Surgery	50 mg daily continuously; 25 mg daily continuously	NA	PD	6 months	Gastrointestinal side effects
Tena, 2018 (36)	M	63	Abdominal	7.5 × 5 cm	Yes	*SDHB*	Surgery	25 mg daily continuously; 37.5 mg daily continuously	PERCIST	PD	2 months	NA
Irwin, 2019 (37)	M	48	Abdominal	7.5 × 6.0 × 4.5 cm	Yes	*SDHB* and *ATRX* (somatic)	Surgery, cyclophosphamide, vincristine, and dacarbazine (CVD)	NA	NA	PD	24 months	NA
Tong, 2019 (38)	F	41	Abdominal	NA	Yes	*SDHB*	Surgery, ^131^I-MIBG	37.5 mg daily continuously	NA	PD	3 months	NA
O**’**Kane, 2019 (16)	14 M, 11 F	Median 50 (17–79)	11 PGL, 14 PCC	NA	22 Yes, 3 No	5 *SDHB*, 1 *SDHA*, 1 *SDHC*, 1 *RET*, 1 *MAX*	16 surgery, 3 chemotherapy, 1 cisplatin/vinorelbine, 1 CVD, 1 carboplatin/etoposide and temozolomide/capecitabine	Sunitinib 50 mg orally, daily for 4 weeks, followed by 2 weeks observation	RECIST	0 CR, 3 PR, 16 SD, 4 PD	Median PFS 13.4 (5.3-24.6)	Fatigue, nausea/vomiting, palmar-plantar erythrodysesthesia syndrome, diarrhoea, hypertension, mucositis, dysguesia, anorexia, thrombocytopenia, AST/ALT increase, anaemia, hypothyroidism, neutropenia, elevated creatinine, hyponatraemia, cardiac ischaemia, cardiomyopathy

M, male; F, female; NA, not available; NS, not specified; PD, progressive disease; SD, stable disease; PR, partial response; CR, complete response; RECIST, Response Evaluation Criteria in Solid Tumors; PERCIST, Positron Emission Tomography Response Criteria in Solid Tumors; PGL, Paraganglioma; PCC, pheochromocytoma.

## Results

**Figure 3:**
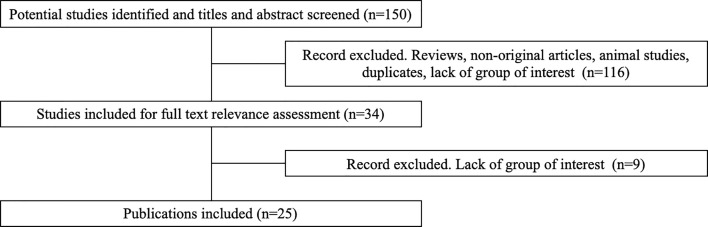
Flow-chart of the literature eligibility assessment process.

Some 150 potentially relevant studies were identified, 116 were excluded on the basis of title and abstract screening. The main reasons for exclusion were not original studies, reviews, animal studies, duplicates, lack of group of interest (treatment with sunitinib). Of the 34 remaining publications, nine were excluded after full text assessment because they did not meet all the eligibility criteria. This process leaded to the selection of 25 publications (**Figure 3**) (16, 20–43).

### Case Series and Case Reports

Data about objective response were available for 23 patients (nine women and 10 men, sex was not available for four patients) (**Table 1**). The patient age range was 20–77 years. Cumulative mean age, and cumulative median age were 45.4 and 47 years, respectively. In all cases disease status at baseline was represented by progressive or inoperable disease. Metastases were present in all patients. Some 14 patients had abdominal PGL, three head & neck PGL, and two pelvic PGL.

Mutation status was examined in 18 patients showing: *SDHB* (n = 15), *MAX* (n = 1), and *SDHC* (n = 1) mutations; one patient had sporadic PGL.

Treatment schedule included sunitinib regimens using 50, 37.5 or 25 mg. Six patients (26.1%) showed progressive disease (PD), four patients (17.4%) stable disease (SD), three patients (13%) partial response (PR), one (4.3%) patient complete response (CR), two patients (8.7%) SD followed by PD, six patients (26.1%) PR followed by PD, and one (4.3%) CR followed by relapse.

Regarding previous therapy, 16 patients underwent surgery before sunitinib treatment, 10 were treated with chemotherapy, six with radiotherapy, five with ^131^I MIBG, three with octreotide, two with PRRT, and one with everolimus. Only two patients started sunitinib as a first-line therapy. Data about objective response in this subset were available for two patients, one patient showed PR followed by PD, and one patient showed SD. In six cases sunitinib was used as first-line systemic therapy after surgery, four patients showed PR followed by PD, and two PD. In one case sunitinib was the first-line systemic therapy after surgery and radiotherapy, the patient showed PR. In any other case sunitinib was prescribed after one or more systemic therapies.

### Clinical Trial

The only clinical trial available in the literature has been published by O’Kane et al. (16) who evaluated the effects of first-line sunitinib treatment (50 mg orally, daily for 4 weeks, followed by 2 weeks observation) in 25 patients (14 men and 11 women) with non-resectable or metastatic progressive PGL (11) or pheochromocytoma (14). Median age was 50 years (range: 17–79 years). Five patients were *SDHB* mutated, one *SDHA*, one *SDHC*, one *RET*, and one *MAX*. The disease control rate was 83% (95% CI: 56–93%), including three patients who achieved a PR, and 16 with SD. The median progression free survival (PFS) was 13.4 months (95% CI: 5.3–24.6) (16) (**Table 1**).

## Discussion

In the present case report, the patient presented bilateral neck PGLs, the right lesion has been removed surgically, however a permanent lesion of the right hypoglossal nerve has occurred. After surgery, the patient still complained dysphagia and pain related to the left PGL. Therefore, given the high surgical risk due to the dimension and the localization of the left lesion, and the patient’s preference, a medical therapy has been proposed.

Current guidelines are lacking therapeutic options for patients with inoperable neck PGLs (13). The established localized treatments include external beam radiotherapy (44), radiosurgery (45), and ablative therapy (46). Whereas systemic therapies include radionuclide therapy with 131I-metaiodobenzylguanidine (MIBG) (47) or peptide receptor radionuclide therapy (PRRT) (48), chemotherapy with cyclophosphamide, vincristine and dacarbazine (CVD) (49), or temozolomide (50). Among molecular targeted therapies the efficacy of TKI as lenvatinib (NCT03008369), cabozantinib (NCT02302833), nivolumab, and ipilimumab (NCT02834013) are currently being evaluated in clinical trials (51). Recently, data from a phase 2 clinical trial provided the rationale for pembrolizumab use in patients with advanced PGLs (52).

Somatostatin receptors (SSTRs) are expressed by PGLs (53, 54), as confirmed by studies investigating ^68^Ga PET-CT role in PGLs diagnosis, staging, and follow-up (17, 55). Therefore, in our case, considering the ^68^Ga DOTATOC uptake SSAs were administrated (lanreotide 120 mg every 28 days). Currently, the efficacy of SSAs in PGLs has not been studied in phase 2–3 clinical trials, nevertheless, in a prospective intervention study, one of four patients with progressive head and neck PGLs treated with octreotide showed a reduced tumor growth (56). In the present case, the SSA was reduced because of side effects, then discontinued because of tumor progression.

Sunitinib has been chosen as a second-line medical therapy after a multidisciplinary consult. Sunitinib is a multitargeted receptor TKI which exerts antiangiogenic and antitumor effects targeting platelet-derived growth factor receptor (PDGFR), vascular endothelial growth factor receptor (VEGFR), KIT, and FLT3 (57). The rationale derived from the high expression of HIF1α, HIF2α, VEGF, and VEGFR arising from HIF dysregulation and hypoxia-inducible target genes’ activation, due to germline mutations of *SDHD* (58). Indeed, the tumorigenesis of PGLs seems partly related to a pseudo-hypoxic drive (59). These data support the rationale of this antiangiogenic therapeutic agent in PGLs.

Recently, O’Kane et al. evaluated the effects of sunitinib treatment on 25 patients with progressive PGL or pheochromocytoma in a phase 2 clinical trial (16). A disease control rate (DCR = SD + PR + CR) of 83% (95% CI: 56–93%) was obtained (16). Notably, three patients who had a PR carried germline mutations of *SDHA*, *SDHB*, and *RET*. Moreover, in four other patients with germline *SDH* mutations (three *SDHB* and one *SDHC*) a prolonged SD was achieved. No patient with *SDHD* mutation was included in the study (16). In our review a significant radiographic DCR was achieved in 34.7% of cases (SD in 17.4%, PR in 13%, and CR in 4.3%). In 39% of patients DCR was followed by PD or tumor relapse. Furthermore, 26.1% patients showed PD.

The discordant results between O’Kane et al. trial and our systematic literature review could be related to the inhomogeneity of the two populations. Indeed, 13 of 23 patients included in our review received two or more lines of therapy before sunitinib, while in the SNIPP trial only one patient received two lines of therapy. Moreover, patients in the SNIPP trial had PGL or pheochromocytoma, while in our literature review patients with pheochromocytomas were not included in the analysis. Furthermore, patients in the two populations received different sunitinib schedules.

The role of sunitinib in these patients could soon become clearer thanks to the First International Randomized Study in Malignant Progressive Pheochromocytoma and Paraganglioma (FIRSTMAPPP) trial, which is investigating the efficacy of sunitinib, at a starting dose of 37.5 mg daily, on the PFS of patients with progressive malignant PGL or pheochromocytoma (NCT01371201). Estimated study completion date of FIRSTMAPPP trial is June 2021.

In our case, the tumor response was early assessed by CEUS, according to previous studies (17). After 20 days of sunitinib therapy a SD was observed, the left lesion was reduced by approximately 10 mm and the right by 4 mm. In the subsequent CEUS evaluation after 56 days both lesions showed stable dimensions. Furthermore, the objective tumor response was accompanied by a clinical improvement of dysphagia and pain, leading to a better quality of life. The treatment has been well tolerated who did not report any specific side effect.

A previous study showed that ^18^F-FDG PET could be a reliable technique to evaluate tumor objective response in sunitinib treated PGLs (24). Indeed, all five *SDHB*-mutated patients with sympathetic PGL who were studied with ^18^F-FDG PET showed a PR or a SD according to RECIST 1.1 criteria (24). In a *SDHB* knockout mouse model of PGL treated with sunitinib, ^18^F-FDG PET was able to detect a transient reduction of FDG uptake and total lesion glycolysis (TLG) during the first two weeks of treatment. However, both SUV and TLG increased after the third week. Metabolic resistance preceded tumor growth which was evident after four weeks of treatment. This evidence suggests that ^18^F-FDG PET could monitor precisely metabolic changes of PGL during an anti-angiogenic treatment and could possibly predict disease progression (60).

In the literature, to the best of our knowledge, among all patients with PGL treated with sunitinib, none was an *SDHD* mutation carrier, and none had a non-metastatic PGL (**Table 1**), making our patient the first case of *SDHD-*related benign PGL treated with sunitinib. Moreover, our patient showed a novel variant of *SDHD* gene, c.16_28del p. Arg6PHEFS*5, which is not described in the literature, neither present in genome (https://gnomad.broadinstitute.org) or in gene-specific databases (https://databases.lovd.nl/shared/genes/SDHD). The abovementioned variant is a small deletion of 13 nucleotides in exon 1 of *SDHD* gene, which determines a nucleotide sequence frameshift, resulting in a new aminoacidic sequence, starting from amino acid 6, with a premature stop after five amino acids. This variant has been classified as pathogenetic (class 5), according to American College of Medical Genetics and Genomics (61).

## Conclusions

In the present paper we report the case of a patient affected by FPGL1 with locally-advanced bilateral neck PGLs. The peculiarity of the case lies in the fact that, to the best of our knowledge, this is the first reported patient with non-metastatic *SDHD*-related PGL treated with sunitinib. The treatment was safe and effective both in terms of tumor objective response and symptomatic relief. Moreover, both the patient and his father carry a novel mutation of *SDHD* gene, associated to the development of PGLs, never described in literature. On the basis of the reported pooled data from our systematic review, sunitinib could be a viable therapeutic option in advanced/progressive/metastatic PGLs, especially in patients with germline mutations. Further trials on the efficacy of sunitinib in FPGL and sporadic PGLs are needed.

## Data Availability Statement

The raw data supporting the conclusions of this article will be made available by the authors, without undue reservation.

## Author Contributions

FS is the first author for this case report and systematic review of literature. EG is the corresponding author that concepted and designed the study. TF, GP, RC, VV, OB, and CV contributed to the data collection, and manuscript, tables, figures preparation. CV, OB, AL, AI, AF, and EG revised critically this work. All authors contributed to the article and approved the submitted version.

## Funding

Ministerial research project PRIN2017Z3N3YC.

## Conflict of Interest

The authors declare that the research was conducted in the absence of any commercial or financial relationships that could be construed as a potential conflict of interest.
